# Serum Metabolic Profiling Reveals Altered Metabolic Pathways in Patients with Post-traumatic Cognitive Impairments

**DOI:** 10.1038/srep21320

**Published:** 2016-02-17

**Authors:** Lunzhao Yi, Shuting Shi, Yang Wang, Wei Huang, Zi-an Xia, Zhihua Xing, Weijun Peng, Zhe Wang

**Affiliations:** 1 Yunnan Food Safety Research Institute, Kunming University of Science and Technology, 650500, Kunming, China; 2 Research Center of Modernization of Traditional Chinese Medicines, College of Chemistry and Chemical Engineering, Central South University, 410083,Changsha, China; 3 Institute of Integrated Medicine, Xiangya Hospital, Central South University, Changsha 410008, P.R. China; 4 Department of Integrated Chinese and Western Medicine, The Second Xiangya Hospital, Central South University, Changsha 410011, P.R. China

## Abstract

Cognitive impairment, the leading cause of traumatic brain injury (TBI)-related disability, adversely affects the quality of life of TBI patients, and exacts a personal and economic cost that is difficult to quantify. The underlying pathophysiological mechanism is currently unknown, and an effective treatment of the disease has not yet been identified. This study aimed to advance our understanding of the mechanism of disease pathogenesis; thus, metabolomics based on gas chromatography/mass spectrometry (GC-MS), coupled with multivariate and univariate statistical methods were used to identify potential biomarkers and the associated metabolic pathways of post-TBI cognitive impairment. A biomarker panel consisting of nine serum metabolites (serine, pyroglutamic acid, phenylalanine, galactose, palmitic acid, arachidonic acid, linoleic acid, citric acid, and 2,3,4-trihydroxybutyrate) was identified to be able to discriminate between TBI patients with cognitive impairment, TBI patients without cognitive impairment and healthy controls. Furthermore, associations between these metabolite markers and the metabolism of amino acids, lipids and carbohydrates were identified. In conclusion, our study is the first to identify several serum metabolite markers and investigate the altered metabolic pathway that is associated with post-TBI cognitive impairment. These markers appear to be suitable for further investigation of the disease mechanisms of post-TBI cognitive impairment.

Cognitive impairment, including loss of consciousness, attention and memory impairments, and/or other alterations of consciousness^1^, is one of the most significant disabilities due to traumatic brain injury (TBI)^2^. Epidemiologic research has indicated that approximately 65% of patients with moderate to severe TBI experience long-term cognitive impairments, and as many as 15% of patients with mild TBI will develop persistent neurological sequelae, including cognitive impairment^3^,^4^. This disability can impose enormous social and economic costs on families and public health systems as a whole^5^. Unfortunately, despite the numerous studies related to this issue, the pathophysiological mechanisms underlying these persistent impairments are still not fully understood^6^. Furthermore, although the published literature, offers potentially useful treatments for cognitive impairments following TBI^7^,^8^, none has been approved by United States Food and Drug Administration (FDA)^1^. This failure in the clinical translation of preclinical studies highlights an urgent need to identify alternative disease mechanisms and relevant biomarkers that can help to completely understand the pathophysiology of the cognitive impairment arising from TBI and to provide a novel route to facilitate a more rational approach to translational research.

Metabolomics is an emerging approach in systems biology; this technique, has recently attracted growing interest in fields such as disease diagnosis, pathology, and toxicology, because it can reveal the characteristic chemical fingerprints that cellular processes leave behind and thus provide often unexpected and unique insights into various biological processes^9^,^10^,^11^,^12^,^13^. For TBI, the superiority of metabolomics in investigation of the metabolic response to TBI has been demonstrated using NMR-based metabolomics^14^,^15^,^16^. More recently, increasing evidence, including data from our previous studies, has demonstrated that serum metabolomics, as determined using GC/MS, has emerged as a powerful profiling approach for discovering novel biomarkers. Thus, this approach can be used to identify the involved pathophysiological processes and elucidate novel physiological and pathological mechanisms of various diseases^17^,^18^,^19^,^20^,^21^.

Nevertheless, there has been no reportof serum metabolomics studies of patients with post-TBI cognitive impairment. Therefore, in this study, GC-MS-based serum metabolomics in combination with pattern recognition methods and pathway analysis was performed in 67 healthy controls, 31 TBI patients without cognitive impairment and 72 TBI patients who presented with cognitive impairment. This study aimed to identify global changes in metabolites, canonical metabolic pathways and associated biomarkers that were perturbed across post-TBI cognitive impairments and thus, generate a better understanding of the pathophysiology of TBI cognitive impairments.

## Results

### Metabolic profiling of TBI patients with cognitive impairment

A GC-MS-based metabolomic approach was employed to determine the serum metabolic disorders of TBI patients with cognitive impairment. As shown in Table 1, 42 metabolites, which were involved in the metabolic processes related to amino acids, carbohydrates, energy, lipids, organic acids and urea, were identified and then qualitatively and quantitatively analyzed in detail. For each metabolite, the statistical significance of the differences between TBI patients with cognitive impairment and controls was calculated separately using the Mann–Whitney U test. Twenty metabolites were significantly altered in TBI patients with cognitive impairment compared with the control group. Among these metabolites, the serum levels of three metabolites, α-hydroxybutyrate, galactose, and trans-4-hydroxyproline, increased strikingly in TBI patients with cognitive impairment compared with the healthy controls, whereas the levels of 17 metabolites (lactate, alanine, N-acetyl glycine, glycerate, serine, pyroglutamic acid, 2,3,4-trihydroxybutyrate, glutamic acid, phenylalanine, glutamine, citric acid, tyrosine, palmitic acid, uric acid, linoleic acid, oleic acid, and stearic acid) decreased markedly (t test, p < 0.05 with a signed t value of “1”).

For qualitative and quantitative analysis of the metabolic profiles of the TBI-positive and TBI-negative groups” or “TBI with cognitive impairment (positive) and TBI without cognitive impairment (negative) groups, we also employed a GC-MS-based metabolomics approach to determine changes in metabolites. For each metabolite, the statistical significance of the differences between the positive and negative groups was calculated separately using the Mann–Whitney U test. Seven metabolites, pyruvate, alanine, serine, phenylalanine, galactose, glutamine and arachidonic acid, were significantly altered in positive group compared with the negative group. The levels of four (serine, phenylalanine, glutamine, and arachidonic acid) of these metabolites increased strikingly in positive group compared with the negative group, while three (pyruvate, alanine, glutamine) of these metabolites decreased markedly (t test, p < 0.05 with a signed t value of “1”).

### Metabolomics-based discrimination model identified the key metabolic disorders of TBI patients with cognitive impairment

**Figure 1:**
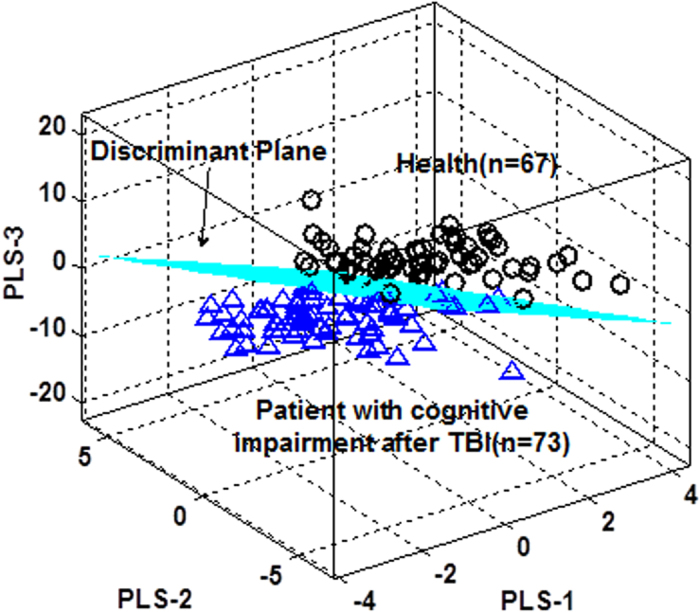
PLS-DA model to discriminate between TBI patients with cognitive impairment and healthy controls.

Partial least squares-discriminant analysis (PLS-DA) was employed to establish a model for discriminating between healthy control subjects and positive group. The autoscaled data set of 42 metabolites was used as input data. A 3-dimensional PLS-DA model, which was constructed using the first three latent variables (PLS-1, PLS-2 and PLS-3) was obtained (Fig. 1). The discriminant plane clearly separated the healthy controls from the TBI patients with cognitive impairment (Fig. 1). The correct rates from the 10-fold cross validation for the healthy controls, the positive group and the total were 94.03%, 84.93%, and 89.29%, respectively. The AUC was 95.62%. These results indicate that the established PLS-DA model is reliable and is good at classifying and discriminating between healthy controls and the positive group.

**Figure 2:**
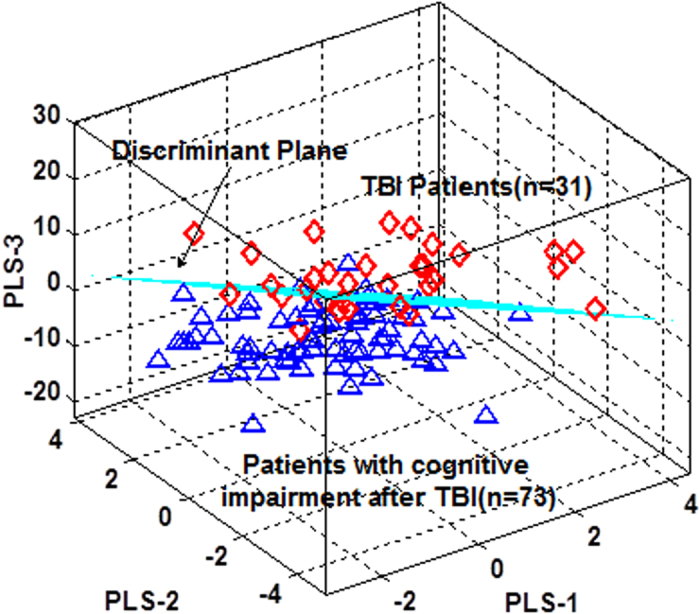
PLS-DA model to discriminate between TBI patients with and without cognitive impairment.

Moreover, PLS-DA was also employed to establish a discrimination model between the positive and negative groups. A 3-dimensional PLS-DA model, which was constructed using the first three latent variables (PLS-1, PLS-2 and PLS-3) was obtained (Fig. 2). The correct rates from 10-fold cross validation for the negative group, the positive group and the total were 83.87%, 71.23%, and 77.04%, respectively. The AUC was 85.02%.

**Figure 3:**
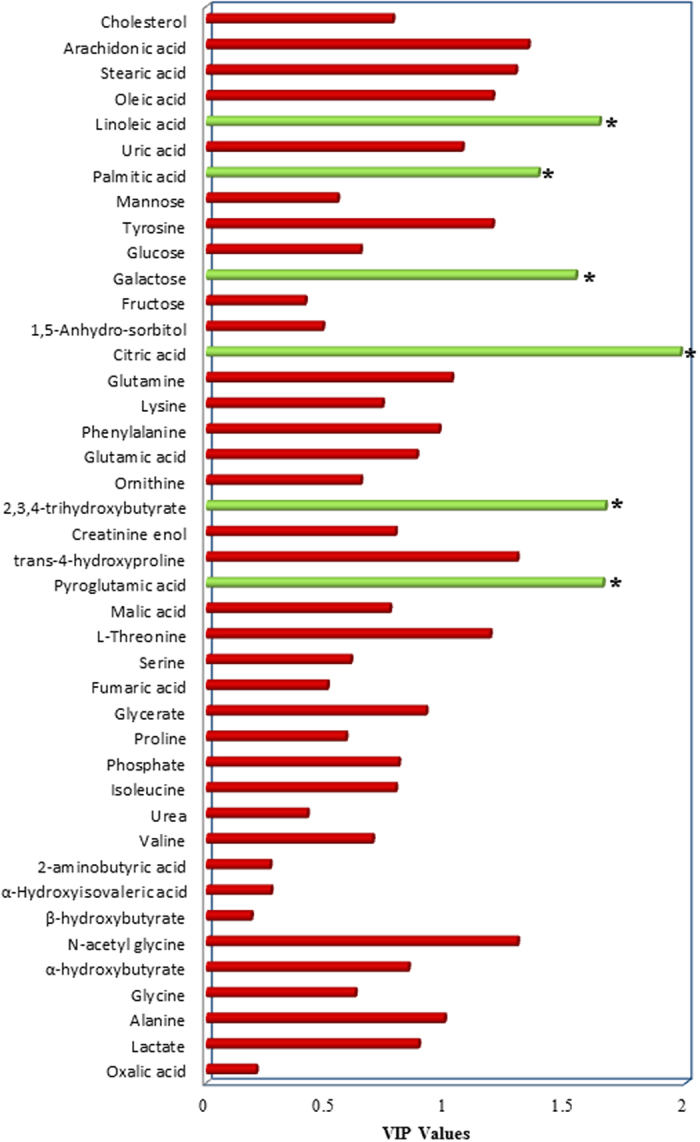
VIP value of each metabolite to discriminate between TBI patients with cognitive impairment and healthy controls. In total, six metabolites (pyroglutamate, 2,3,4-trihydroxybutyrate, citric acid, galactose, palmitic acid, and linoleic acid) were identified.

**Figure 4:**
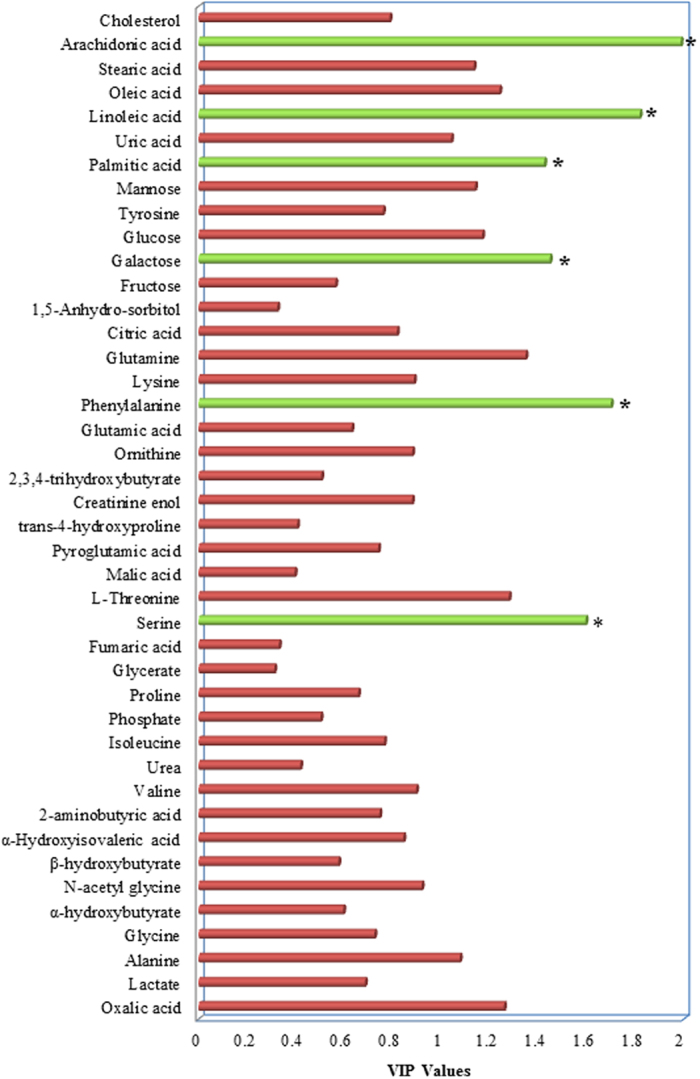
VIP value of each metabolite to discriminate between TBI patients with and without cognitive impairment. In total, six metabolites (serine, phenylalanine, galactose, palmitic acid, linoleic acid, and arachidonic acid) were identified.

**Figure 5:**
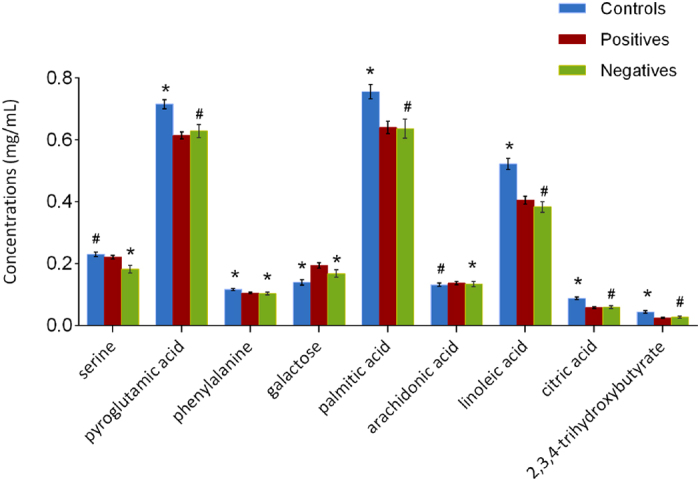
Comparison of the concentrations of nine potential metabolic biomarkers among control, negative and positive groups. Compared with the positive group, * denotes that the difference is considered statistically significant (p < 0.05), and # denotes that the difference is considered no statistically significant (p > 0.05).

After the metabolic discrimination models were established by PLS-DA, variables were selected selection was carried out to identify the candidate biomarkers of TBI patients with cognitive impairment. The variable importance in the projection (VIP) parameter indirectly reflects the correlation of the metabolites with disease and is a widely used method for biomarker selection^22^. Thus, VIP was employed in the present research. For the healthy controls and TBI patients with cognitive impairment, the VIP values of 42 metabolites were calculated and areshown in Fig. 3. A total of six metabolites with significantly altered serum features were selected: pyroglutamic acid, 2,3,4-trihydroxybutyrate, citric acid, galactose, palmitic acid, and linoleic acid (Fig. 3). The VIP values of 42 metabolites for the TBI patients with and without cognitive impairment are shown in Fig 4. A total of six metabolites with significantly altered serum features were selected: serine, phenylalanine, galactose, palmitic acid, linoleic acid, and arachidonic acid (Fig. 4). When considered together, pyroglutamic acid, serine, phenylalanine, galactose, palmitic acid, 2,3,4-trihydroxybutyrate, linoleic acid, citric acid and arachidonic acid could serve as a potential biomarker panel diagnosing of TBI patients with cognitive impairment. Figure 5 shows the result from the comparison of the concentrations of nine potential metabolic biomarkers between the control, negative and positive groups.

### Related metabolic pathways

**Figure 6:**
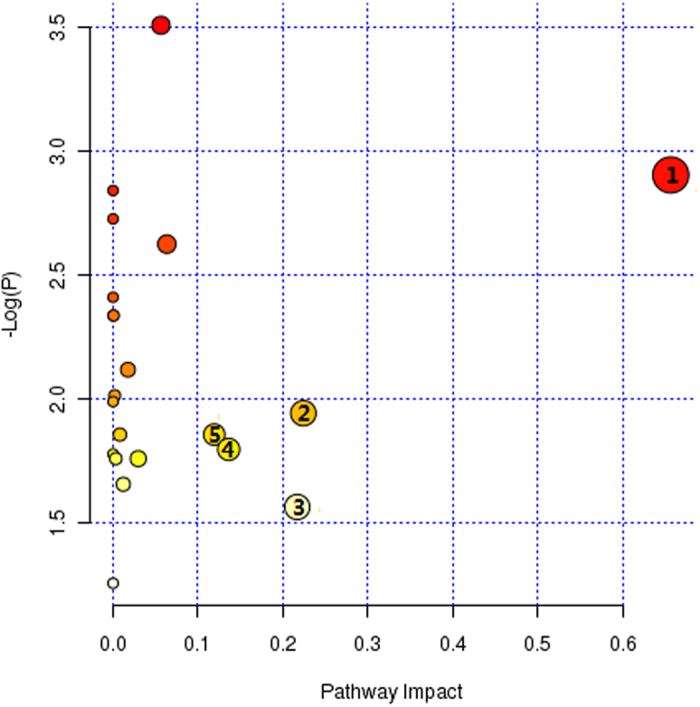
Summary of altered metabolic pathways analysis with MetPA. As shown, five metabolic pathways of importance were disturbed in the serum of TBI patients with cognitive impairment: (1) linoleic acid metabolism; (2) galactose metabolism, (3) arachidonic acid metabolism, (4) glycine/serine/threonine metabolism, and (5) phenylalanine metabolism.

MetaboAnalyst was used to identify the metabolic pathways that were associated with the identified combinations of metabolites, thus indicating which pathways are important for the host response to post-TBI cognitive impairment. Five metabolic pathways of importance (linoleic acid metabolism, galactose metabolism, arachidonic acid metabolism, glycine, serine and threonine metabolism, and phenylalanine metabolism) were disturbed in the TBI patients with cognitive impairment (Fig. 6).

## Discussion

To our knowledge, this is the first study to identify potential biomarkers and unravel the metabolic mechanisms of post-TBI cognitive impairment using serum metabolomics based on GC-MS coupled to both multivariate statistical methods (e.g., PLS-DA) and univariate statistical methods (e.g., the Mann–Whitney U test).

Our data from this study indicate that disease-specific patterns can be identified in the serum metabolomics profile, which distinguishes TBI patients with cognitive impairment from those without cognitive impairment and from healthy controls. In addition, nine serum metabolites (serine, pyroglutamic acid, phenylalanine, galactose, palmitic acid, arachidonic acid, linoleic acid, citric acid,and 2,3,4-trihydroxybutyrate) were selected as potential candidate biomarkers. Furthermore, the KEGG database indicated that these metabolites are mainly associated with metabolic pathways, including those of linoleic acid metabolism, phenylalanine metabolism, glycine/serine/threonine metabolism, arachidonic acid metabolism, galactose metabolism, ascorbate/aldarate metabolism, the citric acid cycle (TCA cycle), and glutathione metabolism; these processes were further associated with the metabolism of amino acids, lipids and carbohydrates. Of these pathways, the linoleic acid metabolism, galactose metabolism, arachidonic acid metabolism, glycine/serine/threonine metabolism, and phenylalanine metabolism pathways were most relevant to post-TBI cognitive impairment revealed, as indicated by the MetaboAnalyst tool; this service is a free, web-based tool that combines the results from powerful pathway enrichment analysis and data from the studied conditions. The most relevant pathways were mainly associated with the metabolism of amino acids, lipids and carbohydrates. The results were used to identify which changes in the metabolic profiles are involved in the pathophysiological mechanism of post-TBI cognitive impairment.

Our findings clearly show that post-TBI cognitive impairment is associated with aberrations in amino acids (serine, pyroglutamic acid, and phenylalanine) and amino acid metabolism (phenylalanine metabolism, glycine/serine/threonine metabolism, and glutathione metabolism). A previous study has also shown that plasma concentrations of amino acids were altered following TBI^23^. As shown in this study, the serum levels of serine, and phenylalanine were significantly higher whereas the pyroglutamic acid level was significantly lower in TBI patients with cognitive impairment than in those without cognitive impairment. Furthermore, it is these results are also consistent with previous reports showing that the levels of serine (L- serine and D- serine) and, phenylalanine were higher in Alzheimer’s disease (AD) patients compared to than in healthy^24^,^25^,^26^. Additionally, the pyroglutamic acid level was decreased significantly in individuals with mild cognitive impairment (MCI) and AD^27^. Of these markers, L-serine and the products of its metabolism have been recognized as essential not only for cell proliferation, but also for specific functions in the central nervous system^28^. D-serine is thought to trigger toxic mechanisms that result in synapse failure in AD, including aberrant N-methyl-D-aspartate receptors (NMDAR) function, increased glutamate release, and impaired synaptic plasticity^26^. Phenylalanine serves as a substrate for the synthesis of neurotransmitters such as dopamine and norepinephrine^25^. Higher serum phenylalanine concentrations are related to immune activation; this increase is a critical factor in the pathogenesis of AD^29^. In addition, pyroglutamic acid could improve the learning and memory capacities in old rat^30^; this effect could also be related to an activation of cholinergic mechanisms^31^.

In terms of lipid metabolism, dyregulated lipid metabolism may be of particular importance for CNS injuries and disorders, including TBI^32^. Furthermore, brain lipid homoeostasis is critical during neurodevelopment and repair after TBI^33^. The serum metabolic profiles that we measured indicate that TBI patients with cognitive impairment had higher arachidonic acid levels and lower palmitic acid and linoleic acid levels. In recent studies, increases in brain arachidonic acid levels were found in an AD model^34^, and the levels of linoleic acid and palmitic acid were significantly lower in AD and amnestic mild cognitive impairment (aMCI) patients than in healthy controls^35^,^36^. Nevertheless, Dhillon *et al.* found that the palmitic acid concentration in the brain is significantly elevated in an experimentally induced model of TB^37^. Furthermore, arachidonic acid, which has proinflammatory activities and could contribute to oxidative stress^38^, was also found to be significantly increased in serum metabolites in TBI subjects^37^,^39^ and was associated with platelet dysfunction following TBI^40^. The elevated levels of palmitic acid could have an important role in the upregulation of secretase (BACE1) and the subsequent amyloidogenic processing of amyloid precursor protein (APP), one of the main characteristic signatures of AD pathology^41^. Linoleic acid, a doubly unsaturated fatty acid and an omega-6 fatty acid, could improve the age-associated decline in cognition and neural function in rats due to the enhancement of protective signaling, the alterations in membrane microstructures, the decreases in inflammation, and the prevention of the accumulation of polyubiquitinated protein aggregates in critical regions of the brain^42^.

Additionally, our observations indicated that post-TBI cognitive impairment was associated with the dysregulation of carbohydrate metabolism, including galactose metabolism, ascorbate and aldarate metabolism, and the TCA cycle. One galactose metabolite, d-galactose is an aldohexose that occurs naturally in the D-form in lactose, cerebrosides, gangliosides, and mucoproteins, and its levels were found to be significantly higher in TBI patients without cognitive impairment than in normal controls. This metabolite is usually used to induce the AD animal model due to its neurotoxicity, which could cause memory impairment, neuroinflammation and neurodegeneration. For carbohydrate metabolites, 2,3,4-trihydroxybutyrate was also found to be decreased in the TBI patients with cognitive impairment. 2,3,4-trihydroxybutyrate is probably derived from glycated proteins or from the degradation of ascorbic acid, and it is a substrate of L-threonate 3-dehydrogenase in the ascorbate and aldarate metabolic pathway. Recently, higher plasma levels of 2,3,4-trihydroxybutyrate were found in patients after acute coronary syndrome^43^. We also observed that citric acid, which is involved in the tricarboxylic acid cycle of the mitochondria, decreased significantly in TBI patients with cognitive impairment; this change was consistent with the change in AD patients^44^.

Recently, numerous epidemiologic studies have indicated that TBI can increase the risk of developing AD^45^,^46^,^47^,^48^,^49^. The association between AD and TBI was further strengthened by clinical and experimental studies^50^,^51^,^52^,^53^,^54^,^55^. Interestingly, our results show that the nine serum metabolites associated with post-TBI cognitive impairment were also changed in AD and that the pattern of changes in these metabolites was similar between post-TBI cognitive impairment and AD. Therefore, we could speculate that TBI patients with cognitive deficits and AD patients may share similar pathophysiological changes at the metabolic level; this similarity provides further evidence for the association between AD and TBI in addition to epidemiologic and pathological evidence.

Although altered metabolites have been identified and their possible role in cognitive impairment following TBI has been investigated, the present study has several limitations. First the metabolomics analysis was only performed using the serum of TBI patients with cognitive impairment was performed. Global metabolic changes in the plasma and CSF of the same individuals with post-TBI cognitive impairment should be determined using a non-targeted metabolomics approach in further studies to more accurately reflect the pathophysiology of cognitive deficits following TBI. Moreover, a correlation has not yet been established between the above-mentioned metabolites and the degree of cognitive impairment. In addition, the present study included only a small number of patients. Additional studies of a larger patient population should be conducted to fully confirm/validate the current metabolite findings. In addition, we only used serum metabolomics that was based upon GC-MS technology to detect the serum metabolic profiles. More metabolomics technologies, such as high performance liquid chromatography–mass spectrometry (HPLC–MS) and nuclear magnetic resonance (NMR), are required to confirm our findings. Finally, the relation between post-TBI cognitive impairment and AD should be further investigated. Because TBI acts as an important epigenetic risk factor for AD^45^, such understanding will help to diagnose the risk of TBI patients of developing AD and design to therapeutic interventions.

## Conclusions

Cognitive impairment following TBI involves a series of complex and dramatic pathophysiological alterations. Therefore, elucidating the pathological changes is a persisting challenge for clinical and basic researchers. Our studies have examined a range of metabolites that represent the metabolic regulation of post-TBI cognitive impairment and illustrated the ability of metabolomics to identify the potential biomarkers of post-TBI cognitive impairment. PLS-DA can facilitate prioritization of the data and greatly increase the probability of identifying metabolites that are causally related to the phenotype of interest. Here, 42 serum differential metabolites were identified. A panel of metabolite markers including serine, pyroglutamic acid, phenylalanine, galactose, palmitic acid, arachidonic acid, linoleic acid, citric acid, 2,3,4-trihydroxybutyrate was selected. This panel was able to discriminate between TBI patients with cognitive impairment and those without cognitive impairment and healthy controls. Post-TBI cognitive impairment was associated with altered metabolism of amino acids, lipids and carbohydrates. When considered together, our studies have elucidated the biomarkers and physiological mechanism of disease in a clinical setting, which suggests that metabolomics is a valuable tool for identifying the molecular mechanisms that are involved in the etiology of post-TBI cognitive impairment and thus novel therapeutic targets.

## Methods

### Ethics statement

All protocols involving the use of human subjects were reviewed and approved by the Ethics Committee of Central South University, Changsha, China (Grant No: 201404366), and all experiments were performed in accordance with relevant guidelines and regulations. Written informed consent was obtained from all patients (or their next-of-kin) enrolled in this study.

### Study subjects and sample collection

All participants presented at the Brain Trauma Specialist Department, department of Encephalopathy of National Key Specialty, and Health Center of the Xiangya Hospital, Central South University, Changsha, China, between February 2014 and December 2014. They are divided into three groups, health controls (67, control group), TBI patients without cognitive deficits (31, negative group) and TBI patients with cognitive deficits (72, positive group). The demographic and clinical chemistry characteristics of enrolled subjects are shown in Table 2.

Eligible TBI patients were between the ages of 14–65 years (inclusive); and had a history of moderate-to-severe TBI (as defined by an initial Glasgow Coma Scale (GCS) score of 12 or less). Upon admission, the overall level of cognitive and behavioral functioning of each TBI patients was assessed using the Rancho Los Amigos Scale (RLAS; also referred to as “Rancho” or the “Levels of Cognitive Functioning Scale”)^56^,^57^. The TBI patients who received scores ranging from Level 1 to Level 8 on this scale were classified into the positive group, and the patients whose level of cognitive and behavioral functioning were better than Level 8 were placed in the negative group.

Individuals were excluded if they met any of the following criteria: (1) serious conditions causing mental disability prior to the TBI, such as developmental handicap (Down’s syndrome), residual disability after previous TBI, confirmed dementia, or serious chronic mental illness (schizophrenia, psychosis or well-confirmed bipolar disorder); (2) severe renal or hepatic impairment; (3) uncontrolled cardiovascular disease; (4) current history of severe abuse of drugs or alcohol; or (5) pregnancy or lactation.

The subjects fasted for at least 12 h before the blood draw. The blood samples were obtained specifically for the purpose of this study and were coded to protect anonymity. Relevant medical data were recorded and coded to match the extracted blood sample. One 3 ml blood serum sample was collected from each enrolled subject. The serum samples were placed in Eppendorf tubes without anticoagulant and were held at 4 °C for 1 h. The sample was centrifuged at 3000 g, for 15 min at 4 °C. The supernatant was the total serum protein. Then, the sample was divided into 0.5 ml aliquots for each tube and stored at−80 °C in a refrigerator for future use.

### GC-MS data acquisition

The process of data acquisition was performed according to previously described procedures^18^. Briefly, each 100-ml serum sample was mixed with 350 μl of methanol, and 50 μl of heptadecanoic acid (dissolved in methanol at a concentration of 1 mg ml^−1^) was added as an internal standard. The mixture was vigorously vortexed for 1 min, and then centrifuged at 16000 rpm for 10 min at 4 °C. The supernatant (400 μl) was transferred to a 5-ml glass centrifugation tube and evaporated to dryness under N_2_ gas. Then, 70 μl of methoxyamine hydrochloride solution (20 mg ml^−1^ in pyridine) was added to the residue, and the mixture was incubated for 60 min at 70 °C. After methoximation, 100 μl of BSTFA derivatization agent was added to the residue and incubated for another 50 min at 70 °C. The final solution was used for GC-MS analysis.

All GC-MS analyses were performed using a gas chromatography instrument (Shimadzu GC2010A, Kyoto, Japan) that was coupled to a mass spectrometer (GC-MS-QP2010) with a constant flow rate of helium carrier gas of 1.0 ml min^−1^. For each sample, 1.0 ml was injected into a DB-5 ms capillary column (30 m × 0.25 mm i.d., film thickness 0.25 mm) at a split ratio of 1:10. The column temperature was initially maintained at 70 °C for 4 min, and then increased at a rate of 8 °C min^−1^ from 70 to 300 °C and held for 3 min. The total GC run time was 35.75 min. The mass spectrometry conditions were maintained as followed: ionization voltage, 70 eV; ion source temperature, 200 °C; interface temperature, 250 °C; full-scan mode in the 35–800 amu mass ranges with 0.2 s scan velocity; and detector voltage, 0.9 kV.

### Identification of endogenous metabolites

All GC-MS data, including retention characteristics, peak intensities, and integrated mass spectra, of each serum sample were used for the analysis. First, the automated mass-spectral deconvolution and identification system (AMDIS software, National Institute of Standards and Technology, Gaithersburg, MD) was employed for peak finding and deconvolution. The NIST Mass Spectral Search Program Version 2.0 and the characteristic ions was used for tentative identification of the structures of the peaks-of-interest in conjunction with the similarity search of the NIST/EPA/NIH Mass Spectra Library (NIST05), which contains 190,- 825 EI spectra for 163,- 198 compounds. For the identification of endogenous metabolites, full scan mass spectra of these metabolites were searched and analyzed using biochemical databases including the Human Metabolome Database (HMDB) (http://www.hmdb.ca/) and the Kyoto Encyclopedia of Genes and Genomes (KEGG) database (http://www.genome.jp/kegg/). Forty-two metabolites were considered to be the main endogenous metabolites. Twenty-eight metabolites were identified from the their corresponding chemical standards. The peak areas of metabolites were compared with that of the internal standards to semi-quantitatively determine the levels of the metabolites. The peak areas were extracted using our custom scripts to generate a data matrix, in which the rows represent the samples and the columns correspond to the peak/area ratios relative to the internal standard in the same chromatogram.

### Statistical analysis

All datasets were autoscaled before PLS-DA. The data matrix of the relative peak areas that were generated from the metabolic profiles was analyzed by PLS-DA to establish any “groupings” with respect to healthy controls, TBI patients with cognitive impairment (positive group) and TBI patients without cognitive impairment (negative group). A 10-fold cross validation was employed to select the optimal number of latent variables and evaluate the predictive ability of the PLS-DA model. In addition, two indexes, the correct rate and the area under the receiver operating characteristic curve (AUC), were compared to evaluate the classification ability of a model.

After the discrimination model was established by PLS-DA, a variable selection procedure was conducted to identify the novel biomarkers. VIP, which is commonly used in metabolomics, was employed.

VIP identifies the importance of each variable *j*; the importance of is reflected by the *w* value from each latent variable (score)., where *w* is the weight from the PLS analysis. The VIP measure *v*_*j*_ is defined as


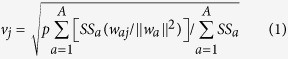


where *p* is the *a*th loading, and SS_*a*_ is the sum of squares corresponding to the *a*th latent variable (score). Hence, the *v*_*j*_ weight is a measure of the contribution of each variable according to the variance corresponding to each PLS latent variable, where (*w*_*aj*_*/*‖*w*_*a*_‖)^2^ represents the importance of the *j*th variable. A higher the value of VIP indicates a higher influence of the corresponding variable. In this study, the variables (metabolites) was were selected based on the VIP values, and the number of metabolites in the combinations was varied from one to eight. We selected the variable combination with the highest AUC value.

All procedures for PLS-DA and other methods were coded in MATLAB 2010 for Windows, and all calculations were performed on an Intel Core i7 processor-based personal computer with 16 G RAM.

### Metabolic pathway analysis

As described in previous studies^19^, the Metabolic Pathway Analysis (MetPA) web tool (http://metpa.metabolomics.ca) was employed to identify the potential affected metabolic pathways on the basis of potential biomarkers; this tool conducts pathway analysis through pathway enrichment analysis and pathway topological analysis. In this work, we selected the Homo sapiens library and use the default ‘Hypergeometric Test’ and ‘Relative-Betweenness Centrality’ algorithms for pathway enrichment analysis and pathway topological analysis, respectively. To identify the most relevant pathways, the impact-value threshold calculated from pathway topology analysis was set to 0.1.

## Additional Information

**How to cite this article**: Yi, L. *et al.* Serum Metabolic Profiling Reveals Altered Metabolic Pathways in Patients with Post-traumatic Cognitive Impairments. *Sci. Rep.* **6**, 21320; doi: 10.1038/srep21320 (2016).

## Figures and Tables

**Table 1:** Qualitative and quantitative analysis of metabolic profiles of control, negative and positive groups.

No.	Metabolites	Concentrations (mg/mL)			T		KEGG	HMDB
		Controls (n = 67)	Positives (n = 73)	Negatives (n = 31)	C-P	N-P		
1	oxalic acid	0.055 ± 0.031	0.054 ± 0.021	0.065 ± 0.026	0	1	C00209	HMDB02329
2	lactate*	3.872 ± 1.298	3.448 ± 0.721	3.466 ± 0.809	1	0	C00186	HMDB00190
3	alanine*	0.412 ± 0.159	0.346 ± 0.136	0.404 ± 0.212	1	1	C00041	HMDB00161
4	glycine*	0.166 ± 0.072	0.179 ± 0.08	0.185 ± 0.093	0	0	C00037	HMDB00123
5	α-hydroxybutyrate	0.080 ± 0.046	0.097 ± 0.043	0.089 ± 0.041	1	0	C05984	HMDB00008
6	N-acetylglycine	0.085 ± 0.036	0.063 ± 0.034	0.052 ± 0.027	1	0	—	HMDB00532
7	β-hydroxybutyrate*	0.129 ± 0.163	0.126 ± 0.199	0.099 ± 0.112	0	0	C01089	HMDB00357
8	α-hydroxyisovaleric acid	0.018 ± 0.010	0.017 ± 0.012	0.021 ± 0.014	0	0	—	HMDB00407
9	2-aminobutyric acid	0.032 ± 0.014	0.033 ± 0.012	0.036 ± 0.016	0	0	C02261	HMDB00650
10	valine*	0.251 ± 0.075	0.246 ± 0.083	0.220 ± 0.090	0	0	C00183	HMDB00883
11	urea	3.847 ± 1.256	3.595 ± 2.037	3.498 ± 2.241	0	0	C00086	HMDB00294
12	isoleucine*	0.257 ± 0.074	0.238 ± 0.061	0.226 ± 0.066	0	0	C00407	HMDB00172
13	phosphate	0.384 ± 0.099	0.385 ± 0.131	0.408 ± 0.383	0	0	C00009	HMDB01429
14	proline*	0.200 ± 0.076	0.201 ± 0.108	0.216 ± 0.143	0	0	C00148	HMDB00162
15	glycerate	0.016 ± 0.006	0.014 ± 0.004	0.014 ± 0.006	1	0	C00258	HMDB00139
16	fumaric acid*	0.015 ± 0.006	0.014 ± 0.007	0.014 ± 0.010	0	0	C00122	HMDB00134
17	serine*	0.230 ± 0.057	0.221 ± 0.054	0.182 ± 0.067	0	1	C00065	HMDB00187
18	L-threonine*	0.246 ± 0.075	0.208 ± 0.05	0.180 ± 0.068	1	0	C00188	HMDB00167
19	malic acid*	0.021 ± 0.012	0.017 ± 0.011	0.018 ± 0.012	0	0	C00149	HMDB00744
20	pyroglutamic acid*	0.715 ± 0.120	0.614 ± 0.101	0.628 ± 0.116	1	0	C01879	HMDB00267
21	trans-4-hydroxyproline	0.043 ± 0.022	0.061 ± 0.036	0.058 ± 0.032	1	0	C01157	HMDB00725
22	creatinine enol	0.063 ± 0.036	0.077 ± 0.048	0.083 ± 0.038	0	0	C00791	HMDB00562
23	2,3,4-trihydroxybutyrate	0.044 ± 0.028	0.025 ± 0.017	0.027 ± 0.019	1	0	C01620	HMDB00943
24	ornithine*	0.076 ± 0.030	0.081 ± 0.041	0.086 ± 0.047	0	0	C00077	HMDB00214
25	glutamic acid*	0.114 ± 0.054	0.097 ± 0.028	0.094 ± 0.026	1	0	C00064	HMDB00148
26	phenylalanine*	0.117 ± 0.025	0.105 ± 0.025	0.103 ± 0.025	1	1	C00079	HMDB00159
27	lysine*	0.079 ± 0.043	0.09 ± 0.049	0.089 ± 0.05	0	0	C00077	HMDB00182
28	glutamine*	0.043 ± 0.023	0.036 ± 0.017	0.036 ± 0.019	1	1	C00064	HMDB00641
29	citric acid*	0.088 ± 0.029	0.058 ± 0.023	0.059 ± 0.027	1	0	C00158	HMDB00094
30	1,5-anhydro-sorbitol	0.319 ± 0.193	0.271 ± 0.219	0.32 ± 0.225	0	0	—	HMDB02712
31	fructose*	0.073 ± 0.042	0.069 ± 0.06	0.063 ± 0.023	0	0	C00095	HMDB00660
32	galactose*	0.139 ± 0.069	0.194 ± 0.072	0.168 ± 0.066	1	1	C01582	HMDB00143
33	glucose*	9.484 ± 2.593	9.055 ± 1.277	8.916 ± 1.07	0	0	C00031	HMDB00122
34	tyrosine*	0.083 ± 0.031	0.064 ± 0.033	0.067 ± 0.033	1	0	C00082	HMDB00158
35	mannose	0.217 ± 0.091	0.205 ± 0.059	0.193 ± 0.037	0	0	C00159	HMDB00169
36	palmitic acid*	0.755 ± 0.193	0.640 ± 0.167	0.636 ± 0.171	1	0	C00249	HMDB00220
37	uric acid	0.277 ± 0.168	0.194 ± 0.132	0.224 ± 0.162	1	0	C00366	HMDB00289
38	linoleic acid*	0.522 ± 0.149	0.405 ± 0.109	0.383 ± 0.098	1	0	C01595	HMDB00673
39	oleic acid*	0.643 ± 0.203	0.547 ± 0.145	0.522 ± 0.146	1	0	C00712	HMDB00207
40	stearic acid*	0.427 ± 0.112	0.368 ± 0.09	0.379 ± 0.098	1	0	C01530	HMDB00827
41	arachidonic acid*	0.132 ± 0.044	0.137 ± 0.043	0.134 ± 0.045	0	1	C00219	HMDB01043
42	cholesterol*	1.545 ± 0.236	1.644 ± 0.382	1.572 ± 0.359	0	0	C00187	HMDB00067

Note: 42 data are presented as mean ± SD. T is the Mann–Whitney U test results between positive, negative and controls; p value of <0.05 is considered statistically significant and signed T value is “1”, otherwise “0”. * Identified by standard substances. C: Controls; P: Positive groups; N: Negative groups.

**Table 2:** Demographic and Clinical Chemistry Characteristics of control, negative and positive groups.

Characteristics	Control	Negative	Positive
Number	67	31	72
Female (%)	32.83 (22/67)	22.58 (7/31)	20.8 (15/72)
Age	52.67 ± 16.71	36.62 ± 16.54	42.48 ± 14.65
FBS (mmol·L^−1^)	5.421±1.416	4.954 ± 1.114	5.48 ± 1.46
HbAlC (%)	1.932 ± 0.818	2.136 ± 0.372	2.05 ± 0.19
TG (mmol·L^−1^)	1.612 ± 1.192	1.735 ± 0.860	1.73 ± 0.79
CHO (mmol·L^−1^)	4.719 ± 1.201	4.491 ± 1.081	5.55 ± 3.94
HDL (mmol· L^−1^)	1.300 ± 0.452	1.227 ± 0.741	1.17 ± 0.52
LDL (mmol·L^−1^)	2.736 ± 0.910	2.774 ± 0.886	3.17 ± 1.15
SBP (mmHg)	121.791 ± 12.246	118.387 ± 17.308	121.75 ± 15.40
DBP (mmHg)	74.299 ± 8.159	77.419 ± 8.249	76.33 ± 11.87
